# Mesenchymal stem cells protect against acetaminophen hepatotoxicity by secreting regenerative cytokine hepatocyte growth factor

**DOI:** 10.1186/s13287-022-02754-x

**Published:** 2022-03-04

**Authors:** Ping Wang, Yan Cui, Jing Wang, Donghua Liu, Yue Tian, Kai Liu, Xue Wang, Lin Liu, Yu He, Yufeng Pei, Li Li, Liying Sun, Zhijun Zhu, Dehua Chang, Jidong Jia, Hong You

**Affiliations:** 1 Liver Research Center, Beijing Friendship Hospital, Capital Medical University, Beijing Key Laboratory of Translational Medicine on Liver Cirrhosis and National Clinical Research Center for Digestive Diseases, No. 95 Yong-An Road, Beijing, 100050 China; 2 BOE Regenerative Medicine Technology Co., Ltd., Beijing, 100015 China; 3 Experimental and Translational Research Center, Beijing Friendship Hospital, Capital Medical University, Beijing Key Laboratory of Tolerance Induction and Organ Protection in Transplantation and National Clinical Research Center for Digestive Diseases, Beijing, 100050 China; 4 Division of Liver Transplantation Surgery, Department of Surgery, Beijing Friendship Hospital, Capital Medical University and National Clinical Research Center for Digestive Diseases, Beijing, 100050 China; 5 Department of Cell Therapy in Regenerative Medicine, University of Tokyo Hospital, Tokyo, 113-8655 Japan

**Keywords:** Mesenchymal stem cells, Acetaminophen, *N*-acetylcysteine, Hepatocyte growth factor

## Abstract

**Background:**

Acetaminophen (APAP) overdose is a major cause of the morbidity of acute liver failure. The current clinically approved treatment for APAP poisoning, *N*-acetylcysteine (NAC), has a limited therapeutic window, and prolonged treatment with NAC delays liver regeneration. Mesenchymal stem cells (MSCs) also have therapeutic effects on APAP-induced mouse liver failure, but whether the effects are comparable to those of NAC has not been determined, and the mechanism still needs further exploration.

**Methods:**

Fasted C57BL/6 mice that received 500 mg/kg APAP were treated intravenously with 300 mg/kg NAC or different amounts of MSCs at 2 h after APAP to investigate survival, hepatocyte necrosis and neutrophil/macrophage recruitment. In vitro co-culture was performed to study the anti-necrotic effects of MSCs on the APAP-injured hepatocyte cell line L-O2.

**Results:**

MSCs dose-dependently rescued the C57BL/6J mice from APAP-induced liver failure, with 87.5% of MSCs (1 × 10^6^) surviving similar to that of NAC (90%). MSC has similar effects on reduced hepatocyte necrosis and granulocytic myeloid-derived suppressor cells (MDSC) infiltration but enhanced the proportion of regenerative monocytic MDSC when compared to NAC. Mechanistically, MSCs attenuate hepatocyte necrosis by secreting hepatocyte growth factor (HGF). When HGF was knocked down, the protective effects of MSCs were reduced on APAP-induced hepatocyte necrosis and mouse liver failure.

**Conclusions:**

MSCs are comparable to NAC against APAP-induced liver failure by secreting HGF with less regenerative retardation concerns, thus facilitating the application of MSCs in clinical therapy for APAP liver failure.

**Supplementary Information:**

The online version contains supplementary material available at 10.1186/s13287-022-02754-x.

## Background

As the most widely used non-prescription analgesic/antipyretic, an overdose of paracetamol (acetaminophen, APAP) can be taken in either deliberately or unintentionally, resulting in acute liver injury and even liver failure. APAP overdose accounts for almost 50% of all deaths from acute liver failure in the USA, the UK and many other Western countries [1–5]. There are several clinical interventions for APAP overdose poisoning, including blocking its absorption by the gastrointestinal tract (activated charcoal to bind APAP, gastric lavage to wash out APAP and ipecacuanha to cause vomiting), removing APAP from the vascular system (using blood filtration equipment) and preventing the formation of toxic metabolites [methionine, cysteamine, dimercaprol or *N*-acetylcysteine (NAC)]. Among the antidotes that may reduce the amount of toxic products, the administration of NAC has been shown to be a safe and effective therapy to improve the survival rate of APAP-induced fulminant hepatic failure in many clinical controlled trials [6, 7]. Until now, NAC has been the only clinically approved antidote recommended and is the most beneficial therapy for APAP overdose patients at risk of liver damage [8, 9]. However, a systematic review of published randomized clinical trials showed that the grade of the evidence quality for NAC is low [10], which may be due to the limited therapeutic window of NAC [11, 12], and prolonged NAC treatment delays liver regeneration by inducing hepatocyte vacuolation [13]. Furthermore, some side effects, including nausea and vomiting, have been reported after either oral or intravenous NAC administration [14–16]. Therefore, there is still an urgent need to develop new therapeutic options for APAP overdose poisoning.

At safe doses, most APAP (90–95%) is metabolized by glucuronosyltransferase or phenolsulfotransferases into non-toxic compounds and removed from hepatocytes by excretion into bile or plasma [17]. Only 5–10% of APAP is metabolized by the hepatocyte cytochrome P450 enzyme Cyp2E1 to generate the reactive and toxic metabolite *N*-acetyl-*p*-benzoquinone imine (NAPQI), which can be detoxified by glutathione (GSH) [17]. APAP overdose leads to GSH depletion and excessive production of NAPQI, which binds to the cysteine residues of proteins to trigger mitochondrial dysfunction and nuclear DNA fragmentation [18], thus resulting in the necrosis of hepatocytes and failure of liver function [16, 19]. As a GSH precursor, NAC can replenish GSH levels to reduce the covalent binding of NAPQI to proteins and scavenge ROS to maintain mitochondrial energy metabolism to restrict hepatocyte necrosis and liver inflammation [17, 20].

Mesenchymal stem cells (MSCs) isolated from bone marrow, adipose tissue, umbilical cord, etc. have demonstrated safety and feasibility with anti-apoptosis, anti-fibrosis and immune modulation effects for many acute and chronic liver diseases [21]. Both human and mouse MSCs have been found to significantly improve the mouse survival rate for APAP-induced fulminant liver failure [22–26], suggesting that the administration of MSCs may be another therapeutic option for APAP poisoning [26]. It has also been revealed that MSCs can inhibit CYP2E1 activity [25], maintain GSH levels [27] and increase antioxidant enzyme activity [28] in APAP-injured rodent livers to reduce hepatocyte necrosis. However, whether the effects of MSCs are comparable to those of NAC has not been considered, and the key cytokines responsible for the therapeutic effects of MSCs have not been revealed. To address this question, we compared the effects of MSCs to NAC on APAP-induced fulminant liver failure and identified the cytokines secreted by MSCs to protect hepatocytes from APAP-induced necrosis, thus providing a preclinical study on the usefulness of MSCs for treating APAP hepatotoxicity.

## Methods

### *Isolation and *in vitro* culture of MSCs*

Healthy pregnant volunteer with no detectable virus infection and no family history of genetic diseases were recruited to this study to donate umbilical cords after childbirth with written informed consent for the GRIPP2 guidelines. This cell-obtaining project was approved by the Ethical Committees of ASIS Hospital Beijing, China (No. LLPJ2017[001]). For mesenchymal stem cell isolation, Wharton’s jelly was isolated, cut into small pieces and digested by collagenase (Sigma-Aldrich, St. Louis, MO, USA). After filtration through 80-mm filter, the cell suspension was centrifuged at 1500 rpm for 5 min to obtain the cells. The cells were cultured and expanded in aMEM (Corning, Manassas, VA, USA) supplemented with 10% foetal bovine serum (Gibco, Auckland, New Zealand) and bFGF (Nanhai Longtime Pharmaceutical, Foshan, China) at 37 °C with 5% CO_2_.

### Flow cytometry for MSC analysis

MSCs were prepared and analysed by surface or intracellular antigen staining according to standard protocols, as described previously [29] using the primary antibodies to detect surface antigens of CD105 (BioLegend, San Diego, CA, USA), CD44 (BioLegend), CD90 (BioLegend), CD73 (BioLegend), CD34 (BioLegend), CD45 (BioLegend) or HLA-DR (BioLegend) and intracellular antigens of HGF (Sigma-Aldrich), EGF (Sigma-Aldrich) or IL-6 (BioLegend). Cell fluorescence was analysed with a FACSAria II (BD Biosciences) using CellQuest software (BD Bioscience).

### Acute liver injury and cell transplantation

Male C57BL/6 J mice with a body weight (B.W.) of 22.0 ± 2.0 g were purchased from Beijing HFK Bioscience Co. (Beijing, China) and maintained under pathogen-free conditions with free access to standard pelleted chow and water. The experimental protocol was approved by the Animal Care and Use Committee of Beijing Friendship Hospital, Capital Medical University (No. 19-2010). The mice were fasted overnight and intraperitoneally administered 0 (*N* = 4), 400 (*N* = 7), 500 (*N* = 7) or 600 mg/kg B.W. (*N* = 7) APAP dissolved in warm saline (15 mg/ml) to optimize the APAP dose, and mouse survival was observed for 7 days [25]. For NAC dose optimization [20], the fasted mice were administered 0 (*N* = 11), 200 (*N* = 8), 300 (*N* = 11) or 400 mg/kg B.W. (*N* = 10) NAC intravenously through the tail vein at 2 h after 500 mg/kg B.W. APAP injection, and mouse survival was observed for 7 days. For survival analysis of MSC treatment, the fasted mice received PBS (100 ml) (*N* = 8) as a negative control, 300 mg/kg B.W. NAC (100 ml) (*N* = 10) as positive control or 5 × 10^5^ (100 ml, *N* = 8), 1 × 10^6^ (100 ml, *N* = 9), 1.5 × 10^6^ (100 ml, *N* = 13), 2 × 10^6^ (100 ml, *N* = 14) MSCs intravenously through tail vein 2 h after 500 mg/kg B.W. APAP injection. Serum from the tail vein of the surviving mice was collected at 24 h post-APAP administration, and mouse survival was observed for 7 days. For tissue analysis and serum NAPQI analysis after MSC treatment, the fasted mice received PBS (100 ml, *N* = 11), 300 mg/kg B.W. NAC (100 ml, *N* = 6) or 1.5 × 10^6^ MSCs (100 ml, *N* = 9) intravenously through the tail vein 2 h after 500 mg/kg B.W. APAP injection, and the tissues and serum were collected 24 h after APAP injection. To analyse the effects of HGF-knockout (HGF^ko^) MSCs, fasted mice received PBS (100 ml, *N* = 9), 1.0 × 10^6^ non-specific gRNA control MSCs (100 ml, *N* = 8) or 1.0 × 10^6^ HGF^ko^ MSCs (100 ml, *N* = 8) or 50 mg/kg B.W. mouse HGF (*N* = 8, Sino Biological, Beijing, China) or 100 mg/kg B.W. mouse HGF (*N* = 8) or 150 mg/kg B.W. mouse HGF (*N* = 8) 2 h after 500 mg/kg B.W. APAP injection, and mouse survival was observed for 7 days.

### Serum NAPQI

Serum NAPQI levels were analysed by mouse NAPQI ELISA kit according to the manufacturer’s instructions (RYBIO, Shanghai, China). Briefly, NAPQI standards (50 ml) and fivefold diluted mouse serum were added to the microelisa strips. Then, HRP-conjugate reagent (100 ml) was added to each well and incubated for 60 min at 37℃. After 5 washes by wash solution, chromogen solution A (50 ml) and chromogen solution B (50 ml) were added to each well and incubated for 15 min at 37℃. After adding stop solution (50 ml), absorbance was measured at 450 nm by microplate reader. The background was corrected by subtracting the final measurement (OD450_final_) obtained for the S0 standard from the OD450_final_ obtained for the standards and samples. Using the corrected measurments, NAPQI levels were determined according to the standard curve and dilution fold.

### Necrosis analysis of APAP-treated L-O2 cells co-cultured with MSCs

L-O2 cells were plated in 6-well plates at 1 × 10^6^ cells/well, and MSCs were plated on chambers adapted to fit in 6-well plates at cell numbers of 5 × 10^5^, 1 × 10^6^, 1.5 × 10^6^ and 2 × 10^6^ at 37 °C overnight. Then, the chambers with MSCs were placed on L-O2 cells and cultured in 20 mM APAP at 37 °C and 5% CO_2_ for 18 h. The chambers with MSCs were discarded, and the remaining supernatant and L-O2 cells in each well were collected and stained with a PE Annexin V Apoptosis Detection Kit I according to the manufacturer’s instructions (BD Pharmingen, Franklin Lakes, NJ, USA). Briefly, after two washes with cold PBS, the cells were resuspended in 1 × Binding Buffer at a concentration of 1 × 10^7^ cells/ml. PE-labelled Annexin V antibodies (1 ml) and 7-AAD (1 ml) were added to 1 × 10^6^ cells and incubated for 15 min at RT in the dark. After adding 400 µl of 1 × binding buffer to each tube, apoptosis/necrosis was analysed by FACSAria II (BD Biosciences) or FACSCalibur flow cytometry (BD Biosciences) using CellQuest software (BD Biosciences).

### Western blotting

Protein extracts were prepared and analysed by Western blotting according to standard protocols, as described previously [29] using the primary antibodies of HGF (Sigma-Aldrich), EGF (Sigma-Aldrich) and IL-6 (BioLegend). Bands were detected using a Molecular Imager ChemiDoc XRS^+^ with Image Lab Software version 3.0 (Bio-Rad, Hercules, CA, USA).

### HGF-knockout MSC preparation

HGF-knockout MSCs were prepared by a two-step CRISPR-Cas9 system. First, 3 × 10^5^ MSCs per well were plated in a 6-well plate, and the cells were infected with lentivirus containing the *Cas9* gene (MOI = 10, SyngenTech, Beijing, China). Four days after infection, the cells were passaged to 60-mm plates and cultured in the presence of blasticidin antibiotic selection at 1.0 µg/mL for 4 days to obtain Cas9-expressing MSCs. Second, 3 × 10^5^ Cas9-expressing MSCs were plated in a 6-well plate, and the cells were infected with lentivirus containing human HGF gRNA (CATCGCCATCCCCTATGCAG) (SyngenTech) or non-specific control gRNA (SyngenTech). Four days after transfection, the cells were passaged to 60-mm plates and cultured in the presence of puromycin antibiotic selection at 1.5 µg/mL for 4 days. The selected cells were expanded in the presence of 0.2 µg/mL puromycin afterwards.

### Transcriptional analysis by RT-PCR

Cells (2 × 10^6^) were used for total RNA extraction, reverse transcription and real-time quantitative polymerase chain reaction (RT-PCR), which were carried out according to the methods described previously [29]. The primers used for amplification are shown in Table 1, and gene transcription levels were calculated relative to GAPDH levels based on the 2-ΔΔCt method [29].

**Table 1:** Primers designed for specific human genes

Name	Accession no	Primer sequence (5′–3′)	Product (bp)
EGF	NM_001963.6	TGTCCACGCAATGTGTCTGAA	133
		CATTATCGGGTGAGGAACAACC	
HGF	NM_000601.6	GCTATCGGGGTAAAGACCTACA	99
		CGTAGCGTACCTCTGGATTGC	
IL-6	NM_000600.5	ACTCACCTCTTCAGAACGAATTG	149
		CCATCTTTGGAAGGTTCAGGTTG	
GAPDH	NM_002046.7	GGAGCGAGATCCCTCCAAAAT	197
		GGCTGTTGTCATACTTCTCATGG	

### Statistics

Sample sizes were chosen based on previous similar experimental outcomes. Data are presented as the mean values ± SD and were analysed for significance using two-tailed Mann–Whitney *U* test with GraphPad Prism 6 software (GraphPad, La Jolla, CA, USA). *P* < 0.05 was indicative of a significant difference.

## Results

### MSC transplantation improves the survival of APAP-treated mice, similar to NAC treatment

**Fig. 1:**
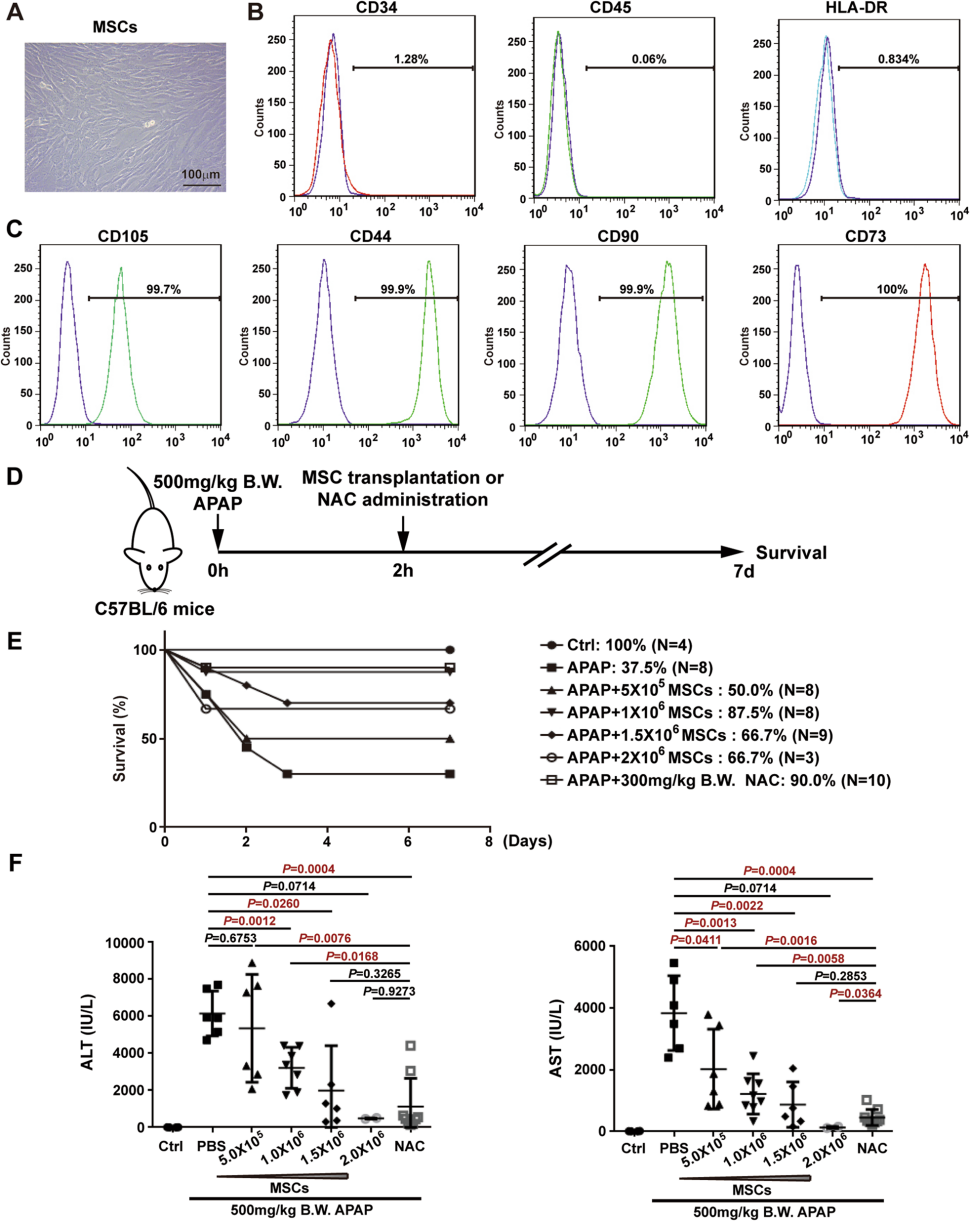
The rescue effects of MSC administration on APAP-induced liver failure are similar to those of NAC. **a** Morphology of in vitro-cultured MSCs. **b** Immunostaining and flow cytometry analysis revealed that MSCs did not express the haematopoietic stem cell marks CD34, CD45 and HLA-DR. **c** Immunostaining and flow cytometry analysis revealed that MSCs expressed CD105, CD44, CD90 and CD73. **d** Schematic representation of the experimental design to determine the effects of MSCs on APAP-induced liver failure. **e** Seven-day survival curve after APAP (500 mg/kg B.W.)-induced liver failure mice treated with PBS or MSCs (5 × 10^5^, 1 × 10^6^, 1.5 × 10^6^, 2 × 10^6^ cells) or NAC (300 mg/kg B.W.). **f** Serum ALT and AST levels at 24 h after APAP challenge. Data are expressed as the mean ± SD, and significant differences between groups are shown in red

Human umbilical MSCs obtained by collagenase digestion showed a fibroblast-like morphology under in vitro culture conditions (Fig. 1a). Immunofluorescence staining and FACS analysis of the immunophenotype of these cells revealed that the positivity rates for the haematopoietic cell markers CD34, CD45 and HLA-DR were less than 2% (Fig. 1b), while the positivity rates for the MSC markers CD105, CD44, CD90 and CD73 were over 99% (Fig. 1c).

Before we investigated the effects of MSCs on mouse survival after APAP challenge, the appropriate APAP dose to induce acute mouse liver failure was determined by the intraperitoneal injection of different doses of APAP (Additional file 1A). As expected, APAP dose-dependently induced mouse death with survival proportions for 400 mg/kg B.W., 500 mg/kg B.W. and 600 mg/kg B.W. of 71.4%, 28.6% and 0%, respectively (Additional file 1A). Therefore, 500 mg/kg B.W. APAP was used in the following experiments to induce mouse liver failure. We also optimized the NAC dose for treating APAP (500 mg/kg B.W.)-induced fulminant liver failure with survival proportions for 200 mg/kg B.W., 300 mg/kg B.W. and 400 mg/kg B.W. of 75.0%, 83.3% and 70.0%, respectively (Additional file 1B). Therefore, 300 mg/kg B.W. NAC was used as the positive control to evaluate the effects of MSCs.

To investigate the therapeutic effects of MSCs on APAP-induced liver failure, different doses of MSCs were administered to APAP (500 mg/kg B.W.)-induced liver failure mice (Fig. 1d). At the time of MSC administration, one mouse (1/9) died immediately after the administration of 1.0 × 10^6^ MSCs, 4 mice (4/13) died after the administration of 1.5 × 10^6^ MSCs, and 11 mice (11/14) died after the administration of 2.0 × 10^6^ MSCs due to pulmonary infarction. Among the remaining mice, the survival proportion of mice increased dose-dependently after the administration of 5 × 10^5^ and 1 × 10^6^ MSCs from 50.0 to 87.5% (Fig. 1e), while a higher dose of MSCs did not further enhance the survival rate (Fig. 1e). The survival proportion was 87.5% in mice that received 1 × 10^6^ MSCs and was similar to the rate of 90.0% in mice that received NAC (300 mg/kg B.W.) (Fig. 1e). Liver damage was monitored by measuring serum ALT and AST levels at 24 h after APAP injection and revealed that the administration of MSCs (≥ 1 × 10^6^) significantly reduced ALT levels, and the administration of MSCs (≥ 5 × 10^5^) markedly reduced AST levels (Fig. 1f). Although a higher number of MSCs (≥ 1.5 × 10^6^) did not further enhance the mouse survival proportion, there was a dose-dependent reduction in the serum levels of ALT and AST from the administration of 5 × 10^5^–2 × 10^6^ MSCs (Fig. 1f). There was no significant difference between MSC (≥ 1.5 × 10^6^) and NAC (300 mg/kg B.W.) administration on ALT levels, and the AST level was significantly lower in MSC (2.0 × 10^6^) administration than that in NAC administration (Fig. 1f). Therefore, MSC administration improves survival and attenuates liver injury in APAP-induced liver failure, which is comparable to the administration of NAC.

### Similar to NAC treatment, MSC administration reduces the necrosis of hepatocytes after APAP challenge

**Fig. 2:**
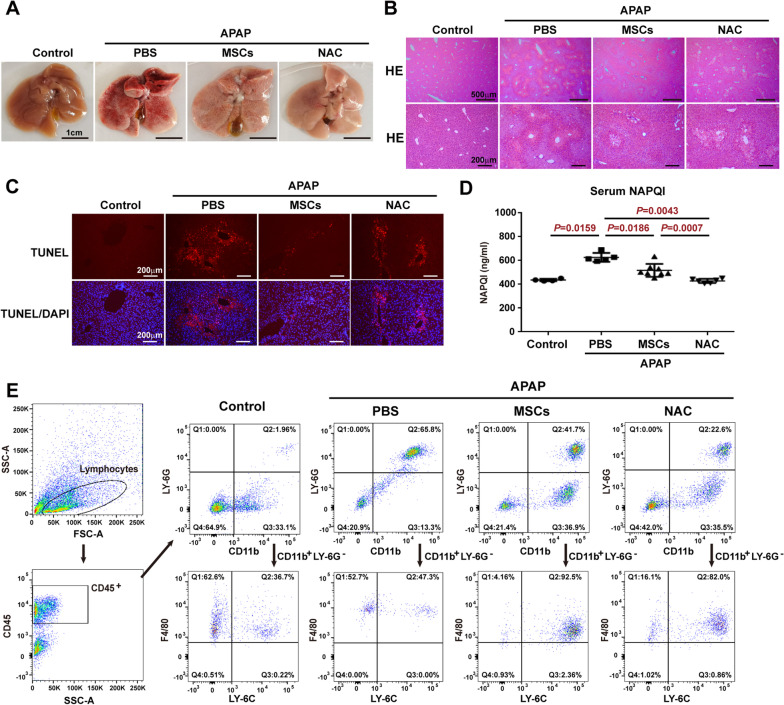
MSCs attenuate hepatocyte necrosis and liver inflammation induced by APAP in a similar tendency to NAC. **a** Representative macroscopic view of livers from control mice or APAP mice administered PBS, MSCs (1.5 × 10^6^) or NAC (300 mg/kg B.W.) at 24 h after APAP challenge (500 mg/kg B.W.). **b** Representative liver sections with HE staining from control mice or APAP mice administered PBS, MSCs (1.5 × 10^6^) or NAC (300 mg/kg B.W.) at 24 h after APAP challenge. **c** Representative liver sections with TUNEL staining from control mice or APAP mice administered PBS, MSCs (1.5 × 10^6^) or NAC (300 mg/kg B.W.) at 24 h after APAP challenge. **d** Serum NAPQI levels analysed by ELISA in control mice or APAP mice administered PBS, MSCs (1.5 × 10^6^) or NAC (300 mg/kg B.W.) at 24 h after APAP challenge. **e** Flow cytometry analysis of the proportion of neutrophils and macrophages recruited to the liver from control mice or APAP mice administered PBS, MSCs (1.5 × 10^6^) or NAC (300 mg/kg B.W.) at 24 h after APAP challenge

To examine histological changes, liver tissue was collected, and hepatocyte necrosis was analysed after the administration of MSCs (1.5 × 10^6^) to treat APAP-induced liver failure. Compared to PBS treatment, both MSC and NAC administration reduced liver congestion at 24 h after APAP administration (Fig. 2a). HE staining showed that although the administration of MSCs and NAC had similar attenuating effects on APAP-induced centrilobular necrosis, the administration of MSCs induced much fewer hepatocyte vacuolation than NAC (Fig. 2b). The extent of centrilobular hepatocyte necrosis and hepatocyte vacuolation was correlated with DNA fragmentation, as demonstrated by TUNEL analysis in Fig. 2c. The serum levels of major hepatocyte toxic metabolite, NAPQI, were increased after APAP administration when compared to control mice, while administration of both MSCs and NAC significantly reduced the serum levels of NAPQI, although the reduction level after MSC treatment was not as much as that after NAC treatment (Fig. 2d). In response to hepatocyte necrosis, the proportion of CD11b^+^Ly6G^+^ granulocytic myeloid-derived suppressor cells (MDSC), a type of immature neutrophils, recruited to the APAP-injured liver was increased compared to that of the control mice, and treatment with MSCs reduced the proportion of CD11b^+^Ly6G^+^ granulocytic MDSC, although not as much as NAC treatment (Fig. 2e). However, MSC treatment enhanced the proportion of CD11b^+^F4/80^int^/Ly-6C^+^ monocytic MDSC with restorative function [30], which was greater than that with NAC treatment (Fig. 2e), suggesting that MSCs recruit more monocytic MDSC to the liver than NAC does.

### MSCs are resistant to APAP-induced injury and protect hepatocytes from APAP-induced injury

**Fig. 3:**
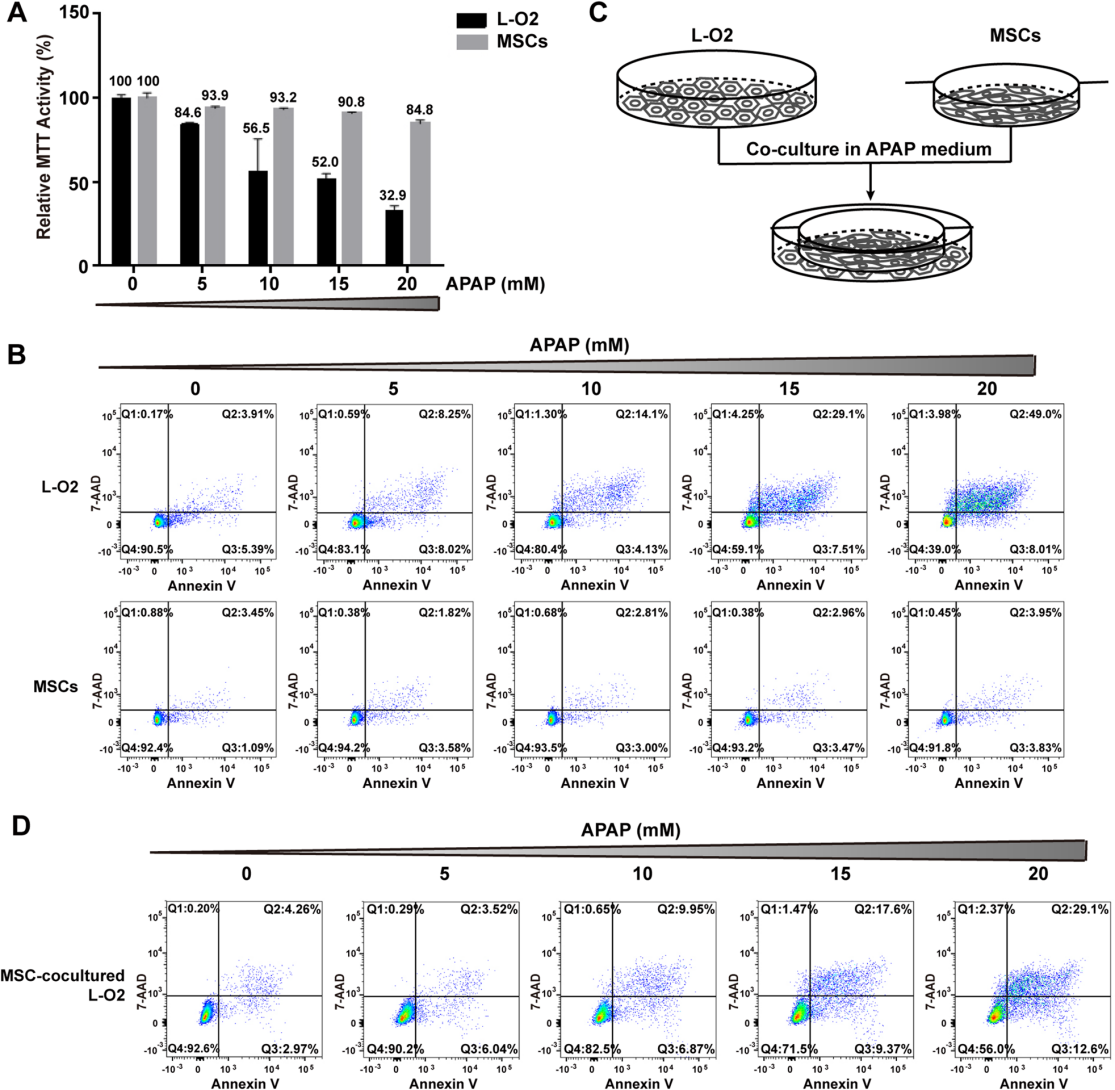
MSCs are resistant to APAP injury and protect L-O2 cells from APAP injury. **a** APAP dose-dependently reduced the viability of L-O2 cells, as determined by MTT analysis, while the reduced viability was not as obvious in MSCs as in L-O2 cells. **b** Annexin V and 7-AAD staining and flow cytometry analysis showed that APAP did not reduce the number of surviving MSCs but significantly reduced the number of surviving L-O2 cells. **c** Schematic representation of the co-culture system of L-O2 cells and MSCs in the presence of APAP. **d** Annexin V and 7-AAD staining and flow cytometry analysis showed that when co-cultured with MSCs (1 × 10^6^), the number of surviving L-O2 cells dose-dependently increased in the presence of APAP (20 mM)

Since MSCs are live cells, the potential ability of APAP to induce MSC necrosis, less than the ability to induce hepatocyte necrosis, is important for their therapeutic efficacy. Different doses of APAP were used to treat MSCs and the human hepatocyte cell line L-O2. Compared to the untreated controls (0 mM APAP), APAP dose-dependently reduced the relative MTT activity of L-O2 cells by 32.9% and of MSCs by 84.8% at 20 mM APAP (Fig. 3a). Flow cytometry analysis of apoptotic/necrotic cells by Annexin V and 7-AAD revealed that APAP did not lower the proportion of surviving MSCs (Fig. 3b lower panel) but dose-dependently reduced that of L-O2 cells (Fig. 3b upper panel), suggesting that MSCs are more resistant to APAP injury than hepatocytes and that the reduction in cell viability based on MTT is due to growth inhibition rather than necrosis induction. When L-O2 cells were co-cultured with 1 × 10^6^ MSCs (Fig. 3c), the proportions of surviving L-O2 cells at different APAP doses were all higher than those of L-O2 cells cultured alone (Fig. 3d), suggesting that MSCs can protect L-O2 cells from APAP-induced necrosis.

### MSCs have similar survival-promoting effects to NAC

**Fig. 4:**
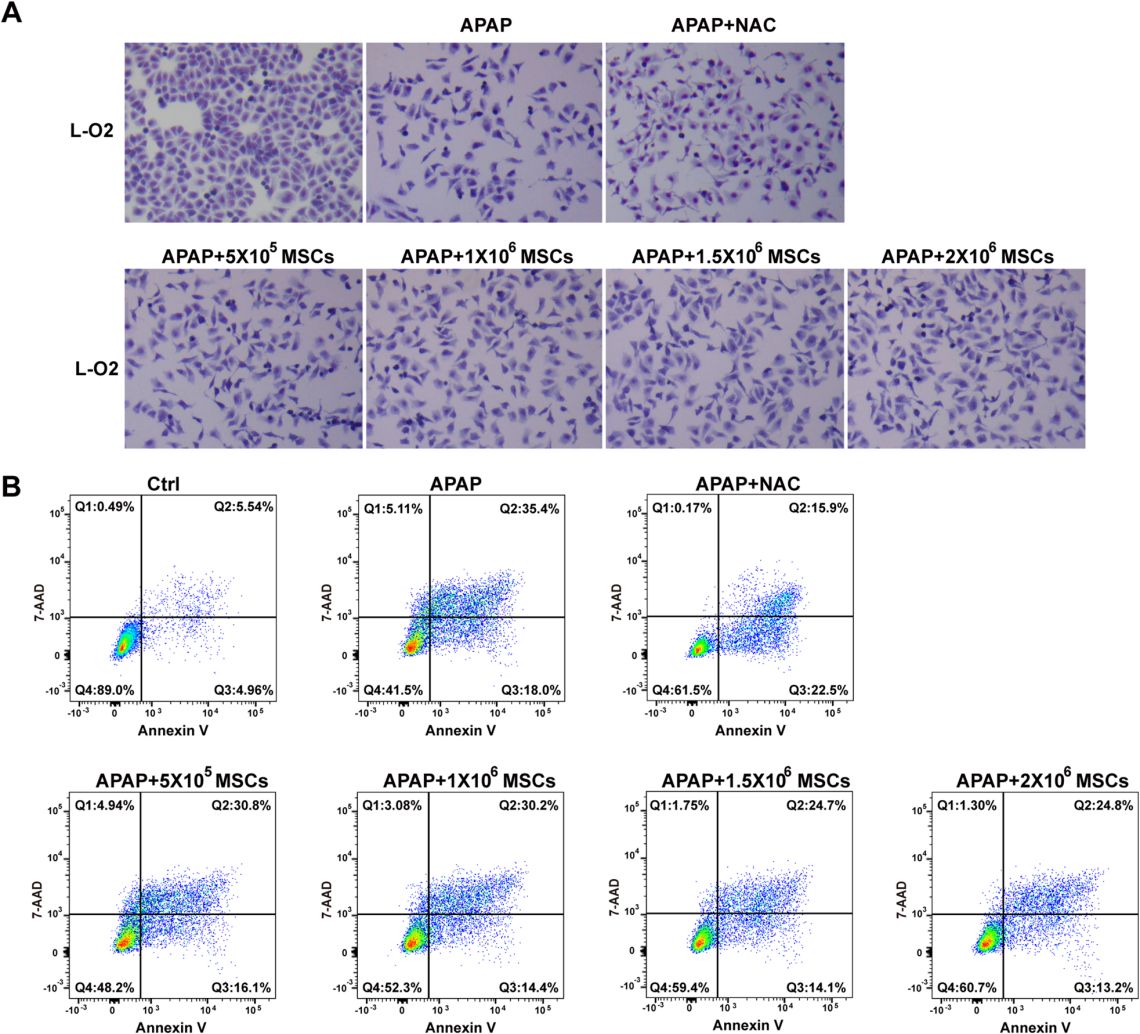
MSCs dose-dependently protect L-O2 cells from APAP-induced necrosis. **a** Giemsa staining of the surviving L-O2 cells after treatment with NAC (6 mM) or co-culture with different numbers of MSCs (5 × 10^5^, 1 × 10^6^, 1.5 × 10^6^, 2 × 10^6^ cells) in the presence of APAP (20 mM). **b** Annexin V and 7-AAD staining and flow cytometry analysis showed that MSCs dose-dependently increased the proportion of surviving L-O2 cells in the presence of APAP (20 mM), and the survival rate of L-O2 cells co-cultured with 2 × 10^6^ MSCs was similar to that of the cells treated with NAC (6 mM)

To compare MSCs to NAC, the optimal NAC dose to protect L-O2 cells from necrosis was analysed by MTT. As shown in Additional file 2, 6 mM NAC showed the highest relative cell viability based on MTT in the presence of 20 mM APAP. Therefore, 6 mM NAC was used as the positive control NAC concentration to protect L-O2 cells from APAP injury. In the presence of 20 mM APAP, MSCs at 5 × 10^5^, 1 × 10^6^, 1.5 × 10^6^ and 2 × 10^6^ cells showed a dose-dependent tendency to increase the survival of L-O2 cells based on Giemsa staining (Fig. 4a), and flow cytometry analysis revealed that the survival proportion of L-O2 cells increased from 48.2 to 60.7% compared to that of non-co-cultured L-O2 cells (41.5%) (Fig. 4b). More importantly, the survival proportion of L-O2 cells co-cultured with 2 × 10^6^ MSCs was 60.7%, which was comparable to 61.5% of the positive control, 6 mM NAC. Therefore, MSCs exerted survival-promoting effects on APAP-injured hepatocytes similar to those of NAC.

### MSCs attenuate APAP-induced necrosis of L-O2 cells by secreting HGF

**Fig. 5:**
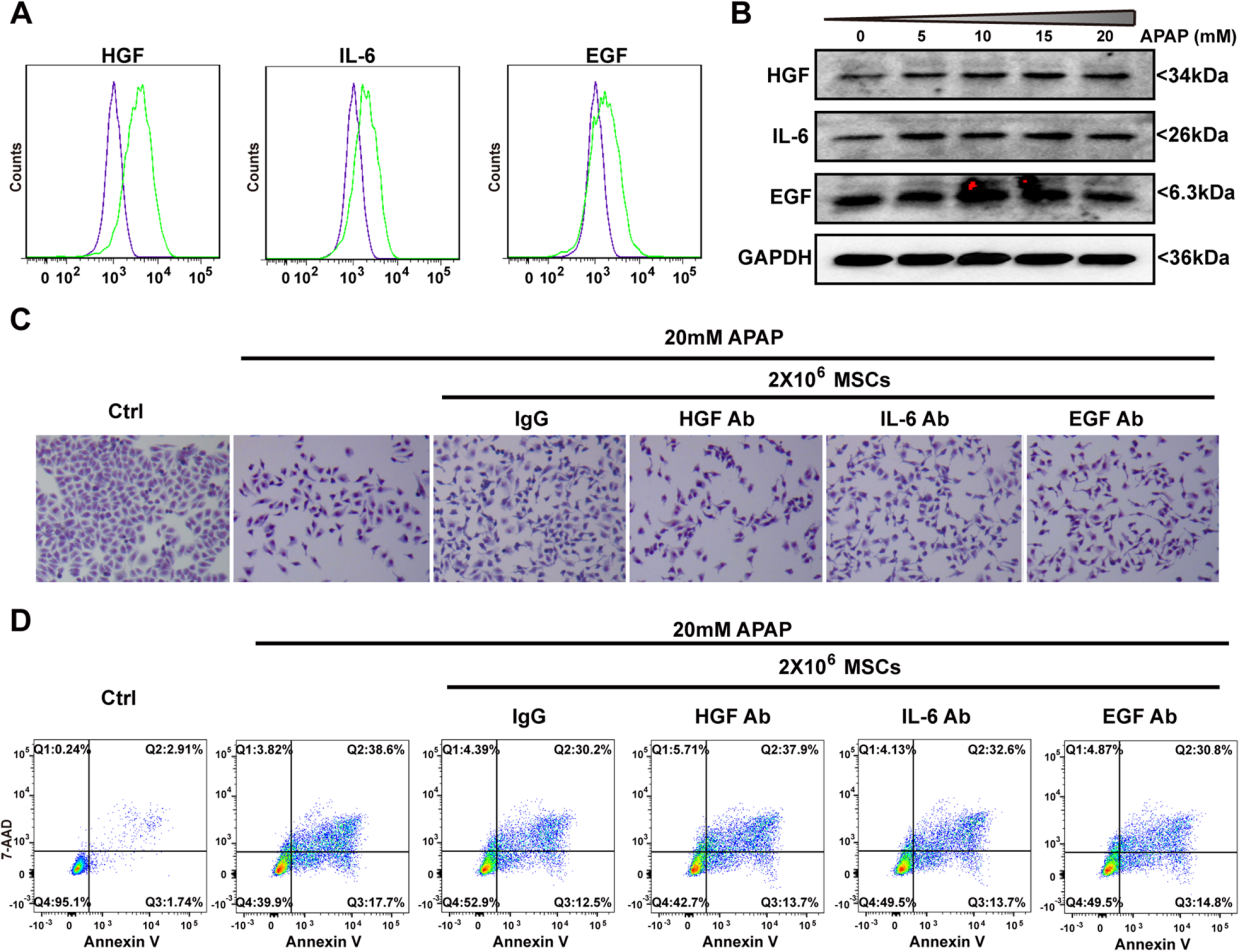
HGF is an essential cytokine of MSCs to protect L-O2 cells from APAP injury. **a** Intracellular staining and flow cytometry analysis of HGF, IL-6 and EGF in MSCs. **b** Western blot analysis showed the sustained expression of HGF, IL-6 and EGF in APAP-treated MSCs. **c** Giemsa staining of the surviving L-O2 cells co-cultured with MSCs and neutralizing antibodies against HGF, IL-6 and EGF in the presence of APAP (20 mM). **d** Annexin V and 7-AAD staining and flow cytometry analysis showed that HGF neutralization antibodies blocked the anti-necrotic effects of MSCs on APAP-treated L-O2 cells compared to IL-6 or EGF neutralization antibodies

It has been reported that MSCs regenerate or repair injured tissue or cells by producing trophic factors [21]. Among the trophic factors secreted by MSCs, HGF, EGF and IL-6 are cytokines that affect hepatocyte survival and/or proliferation [21]. Intracellular staining and flow cytometry analysis showed that MSCs expressed HGF, IL-6 and EGF (Fig. 5a). There was a trend of dose-dependent increases in HGF, EGF and IL-6 expression in the presence of APAP (Fig. 5b). When neutralizing antibodies were added to the co-culture system with L-O2 cells and MSCs, the survival proportions of MSCs in the presence of HGF antibodies, EGF antibodies, IL-6 antibodies and IgG control antibodies were 42.7%, 49.5%, 49.5% and 52.9%, respectively (Fig. 5c, d). This suggests that all three kinds of antibodies blocked the survival-promoting effects of MSCs on APAP-treated L-O2 cells to some extent, but the blocking effects of HGF antibodies were stronger than those of IL-6 antibodies or EGF antibodies (Fig. 5c, d). Therefore, HGF is the main cytokine secreted by MSCs that protects hepatocytes from APAP-induced necrosis.

### Knocking out HGF resulted in the failure of MSCs to rescue APAP-injured mice

**Fig. 6:**
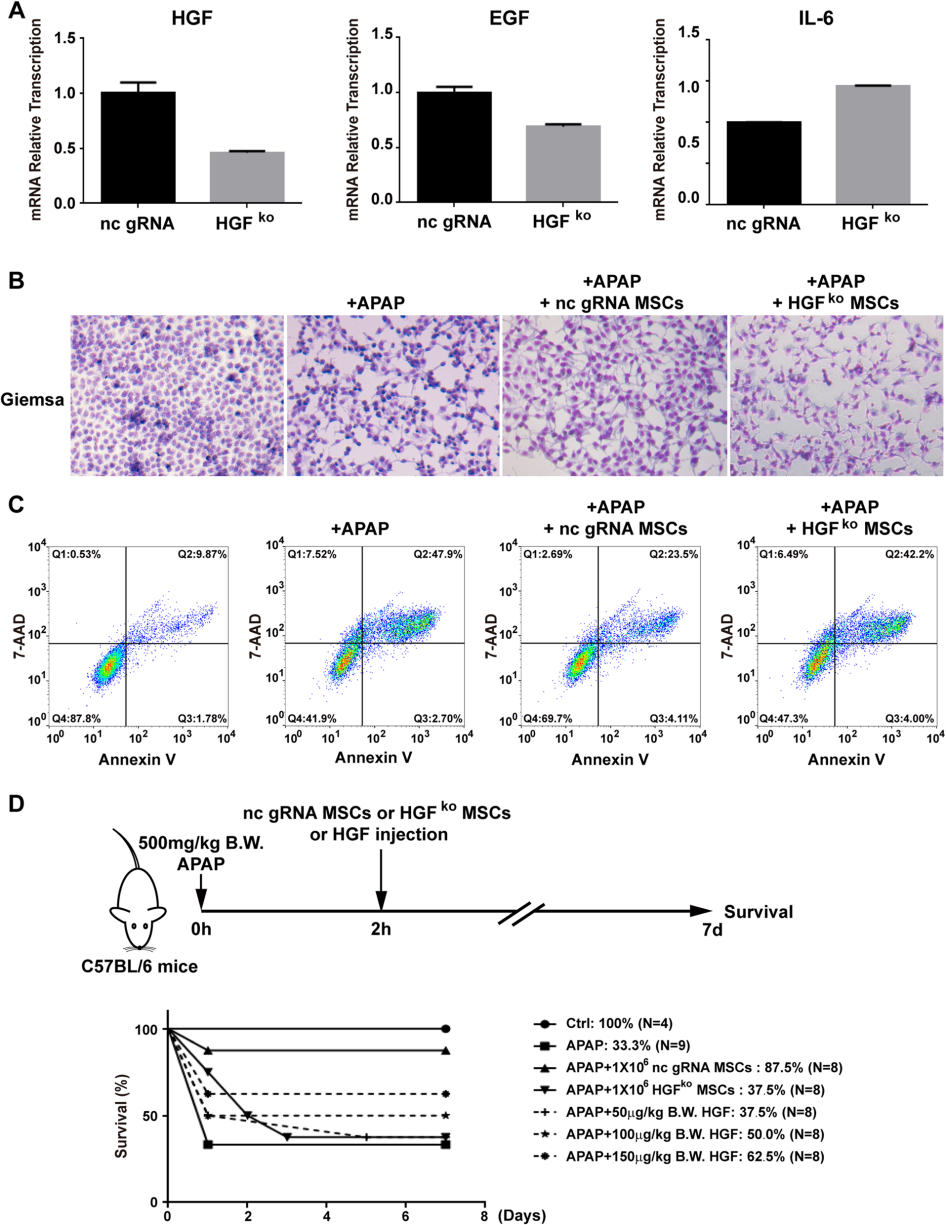
Knockout of HGF abrogates the anti-necrosis effects and therapeutic effects of MSCs on APAP-induced L-O2 cell injury and mouse liver failure. **a** Knockout of HGF by CRISPR-Cas9 reduced HGF transcription. **b** Giemsa staining of the surviving L-O2 cells co-cultured with HGF^ko^ MSCs and non-specific gRNA control MSCs. **c** Annexin V and 7-AAD staining and flow cytometry analysis showed that the knockout of HGF reduced the anti-necrosis effects of MSCs on APAP-treated L-O2 cells compared to non-specific gRNA control MSCs. **d** Seven-day survival curve after APAP (500 mg/kg B.W.)-induced liver failure mice were treated with PBS or HGF^ko^ MSCs (1 × 10^6^) or non-specific gRNA control MSCs (1 × 10^6^) or different concentrations of HGF (50 mg/kg B.W., 100 mg/kg B.W. and 150 mg/kg B.W.)

To confirm that HGF is an essential cytokine of MSCs to protect hepatocytes from APAP injury and rescue mice from APAP-induced liver failure, HGF was knocked out by CRISPR-Cas9 in MSCs. However, single clonal HGF^ko^ MSCs were not successfully expanded in vitro, so we used MSCs after puromycin selection as gRNA control MSCs and HGF^ko^ MSCs. RT-PCR results showed that the transcription level of HGF was approximately half of that in the non-specific gRNA control cells (Fig. 6a), while the transcription level of EGF and IL-6 was approximately 0.7- and 1.4-fold, respectively, of that in the non-specific gRNA control cells (Fig. 6a). When HGF-knockout MSCs were co-cultured with APAP-treated L-O2 cells, the survival proportion of L-O2 cells co-cultured with HGF^ko^ MSCs was 47.3%, which was much lower than the survival proportion of 69.7% for non-specific gRNA control cells (Fig. 6b, c), suggesting that knocking out HGF reduces the protective effects of MSCs on APAP-induced cell necrosis. When transplanted into APAP-challenged mice (Fig. 6d), the survival proportion of APAP-challenged mice was 37.5% after the transplantation of HGF^ko^ MSCs, which was significantly less than the survival proportion of 87.5% observed after the transplantation of non-specific gRNA control MSCs. As positive control, mouse HGF from 50 to 150 μg/kg B.W. rescued the mice from APAP-induced death dose-dependently with survival rate increase from 37.5 to 62.5%, yet the survival rate of HGF (150 μg/kg B.W.) administration was still lower than that of nc gRNA MSC transplantation (Fig. 6d). Therefore, these data suggest that HGF is the essential factor of MSCs that contributes to protecting hepatocytes from APAP-induced liver failure.

## Discussion

Previous studies find the therapeutic effects of MSCs on APAP-induced liver failure [22–26] by inhibiting CYP2E1 activity [25], maintaining GSH levels [27] and increasing antioxidant enzyme activity [28]. Compared to previous studies, there are two novelties of this study. One is that the efficacy of MSC administration to treat APAP injury is similar to that of the clinical used drug, NAC and MSCs induce less hepatocyte vacuolation than NAC. The other is that HGF, a hepatocyte growth stimulatory cytokine, is the major cytokine by which MSCs protect hepatocytes from APAP injury and rescue mice from APAP-induced liver failure. Therefore, administration of MSCs may overcome the problems of regeneration retardation and hepatocyte vacuolation after prolonged NAC treatment, thus serving as a new therapeutic option for APAP overdose poisoning.

For translational consideration, we select human MSCs developed according to GMP guidelines to treat mouse liver injury for obtaining preclinical data of human MSCs as a drug for future clinical research. It has been found that xenotransplantation of human MSCs show therapeutic effects on APAP-induced mouse liver failure [28, 31]. Furthermore, cultured human MSCs only express low levels of MHC class I and do not express MHC class II or co-stimulatory molecules, including B7-1, B-72 and CD40 [32–34], which results in low immunogenicity of MSCs and makes them eligible for allogeneic transplantation [35]. There may be immunological effects in this xenogeneic cell therapy, but if we could get therapeutic effects under these host immunological rejection condition, we could expect a more effective therapeutic outcome during allotransplantation.

There are two essential aspects for evaluating the efficacy of new therapeutic candidates at the preclinical stage: dose-dependent therapeutic effects to treat diseases and at least comparable therapeutic effects to clinically used drugs. For dose-dependent effects, we found that L-O2 cells co-cultured with different numbers of MSCs showed a dose-dependent increase in the proportion of surviving cells in the presence of APAP, confirming the protective effects of MSCs against APAP-induced L-O2 cell necrosis. More importantly, human umbilical cord MSCs (from 1 × 10^5^ to 5 × 10^5^ cells) in APAP mice dose-dependently increased the survival rate of 750 mg/kg B.W. APAP-challenged BALB/c mice, and no further increase was observed in mice transplanted with 1 × 10^6^ MSCs [28]. Similarly, we found dose-dependent therapeutic effects of MSCs (from 5 × 10^5^ to 1 × 10^6^ cells) on 500 mg/kg B.W. APAP-challenged C57BL/6J mice, and no further increase was observed in mice transplanted with 1.5 × 10^6^ or 2.0 × 10^6^ MSCs. Although different mouse subtypes and different APAP doses were used for liver failure induction, both studies observed the dose-dependent therapeutic effects of human MSCs on APAP-induced mouse liver failure.

As an antidote recommended for APAP overdose therapy, NAC has been confirmed to be effective in animal models [20, 36] and clinical trials [6, 7]. Clinically, intravenous NAC administration improves patients with fulminant hepatic failure after paracetamol overdose from 20% (*N* = 25) to 48% (*N* = 48) with three regimens of 150 mg, 50 mg and 100 mg per kg B.W. NAC in 5% dextrose over 15 min, 4 h and over 16 h, with approximately 30% more patients surviving [6]. Human umbilical cord-derived MSCs (5 × 10^5^) increased the survival proportion of 750 mg/kg B.W. APAP-challenged mice from 0 to 40% [28], mouse omentum adipose-derived MSCs (1 × 10^6^) rescued the survival of 600 mg/kg B.W. APAP-challenged mice from 60 to 90% [25], and our human umbilical cord-derived MSCs (1 × 10^6^) rescued survival levels from 37.5 to 87.5% for 600 mg/kg B.W. APAP-challenged mice, which suggests a 30–40% increase in the rescue effects of human MSCs on mouse APAP-induced liver failure. In comparison to NAC, we found that the rescue survival level 87.5% of 1 × 10^6^ MSCs were similar to 90.0% of NAC after administration to the mice at 2 h after APAP injection. Our in vitro studies also confirmed the similar survival-promoting effects of 2 × 10^6^ MSCs to the optimized NAC dose on APAP-treated L-O2 cells. Although the ALT and AST levels of mice treated with 1 × 10^6^ MSCs were higher than those of NAC-treated mice, the ALT and AST levels of mice administered 1.5 × 10^6^ or 2 × 10^6^ MSCs were not significantly different from those of NAC-treated mice. These data further confirm the dose-dependent effects of MSCs and suggest the urgent need to optimize the therapeutic dose of MSCs for APAP-induced liver failure. Therefore, MSCs fulfil the criteria of a new candidate for treating APAP-induced liver failure with dose-dependent therapeutic effects and comparable effects to the clinically used drug NAC.

Hepatocyte vacuolation to delay liver recovery is the major obstacle for NAC prolonged clinical usage to treat APAP-liver failure [13]. In contrast to NAC, MSCs mainly exert therapeutic effects by secreting cytokines to promote hepatocyte proliferation and prevent apoptosis [21]. Among these cytokines, HGF has been revealed to be increased in the serum of MSC-treated APAP-challenged mice [28]. We found that among the three major hepatocyte survival/growth stimulating factors secreted by MSCs, HGF had more anti-necrosis effects on APAP-treated L-O2 cells than EGF or IL-6. More importantly, the rescue effects of HGF-knockout MSCs on the survival of APAP-challenged mice decreased to half of the level of control MSCs, further confirming the essential function of HGF in the therapeutic effects of MSCs. Since HGF-knockout MSC clones were not successfully expanded in vitro, HGF may be an important cytokine that supports the growth of MSCs. Considering that the mechanism through which MSCs affect APAP-induced liver failure involves secreting HGF to rescue hepatocytes from death and promote their proliferation, transplanting MSCs may avoid hepatocyte vacuolation and impeded liver regeneration after prolonged NAC treatment, serving as an additional candidate for APAP overdose patients.

We understand that safety is the most important aspect of drug development, and we noticed that some mice died immediately after the administration of MSCs in a dose-dependent manner. There may be two explanations for this severe adverse event. One is that the MSCs used in these experiments were isolated from humans, and human cells are twice the size of mouse cells [37], thus increasing the risk for pulmonary infarction. The other is that since MSCs were resuspended in 100 ml PBS at the desired concentration for tail vein transplantation, as the cell number used for transplantation increased, the concentration of cell suspension administered to the mice in a very short period of time also increased. For clinical usage, the transplantation of human MSCs to treat human diseases and the intravenous infusion of MSCs at lower cell concentrations and over a relatively long time period may reduce the risk for pulmonary infarction, and many clinical trials have confirmed no critical infusion-related effects of transplanting allogenic MSCs for HBV-related acute-on-chronic liver failure [38] and liver cirrhosis [39]. Therefore, transplanting human allogenic MSCs to humans may not lead to the severe adverse events that were observed in mice.

## Conclusions

In summary, MSCs exert anti-necrosis effects on APAP-injured hepatocytes to increase the survival of APAP-challenged mouse liver failure by secreting HGF, and the efficacy of transplanting MSCs is similar to those of the clinically used drug NAC. Since HGF is a growth stimulatory cytokine for hepatocytes, administration of MSCs may overcome the regeneration retardation and hepatocyte vacuolation effects of NAC after prolonged treatment, thus serving as a new therapeutic candidate for future clinical use of APAP overdose.

## Supplementary Information

**Additional file 1:** Optimize the dose of APAP for liver failure and the dose of NAC for treating APAP-induced liver failure. **A** Schematic representation of the experiment design and seven-day survival curve to determine the dosage for APAP-induced liver failure in mice. **B** Schematic representation of the experiment design and seven-day survival curve to optimize the NAC dosage to treat APAP-induced liver failure in mice.

**Additional file 2:** MTT analysis to optimize the NAC dose for blocking APAP-induced L-O2 cell necrosis.

## Data Availability

The datasets used and/or analysed in this study are available from the corresponding author on reasonable request.

## Abbreviations

NAC: *N*-Acetylcysteine
APAP: Acetaminophen
NAPQI: *N*-Acetyl-*p*-benzoquinone imine
GSH: Glutathione
MSCs: Mesenchymal stem cells (MSCs)
HGF: Hepatocyte growth factor
EGF: Epidermal growth factor
IL-6: Interleukin-6
