# Supplementary material for: Mesenchymal stem cells protect against acetaminophen hepatotoxicity by secreting regenerative cytokine hepatocyte growth factor
Source: Stem Cell Res Ther. 2022 Mar 4;13:94. doi: 10.1186/s13287-022-02754-x (PMC8895877; doi:10.1186/s13287-022-02754-x)
Supplement: Supplementary file 1 — Additional file 1: Optimize the dose of APAP for liver failure and the dose of NAC for treating APAP-induced liver failure. A Schematic representation of the experiment design and seven-day survival curve to determine the dosage for APAP-induced liver failure in mice. B Schematic representation of the experiment design and seven-day survival curve to optimize the NAC dosage to treat APAP-induced liver failure in mice. [file 13287_2022_2754_MOESM1_ESM.docx]

**Mesenchymal Stem Cells Protect Against Acetaminophen Hepatotoxicity by Secreting Regenerative Cytokine Hepatocyte Growth Factor**

Ping Wang^1#^, Yan Cui^1#^, Jing Wang^2^, Donghua Liu^2^, Yue Tian^3^, Kai Liu^3^, Xue Wang^1^, Lin Liu^1^, Yu He^1^, Yufeng Pei^1^, Li Li^1^, Liying Sun^4^, Zhijun Zhu^4^, Dehua Chang^5^*, Jidong Jia^1,3^*, Hong You^1,3^*

^1^ Liver Research Center, Beijing Friendship Hospital, Capital Medical University, Beijing Key Laboratory of Translational Medicine on Liver Cirrhosis & National Clinical Research Center for Digestive Diseases, Beijing 100050, China.

^2^ BOE Regenerative Medicine Technology Co., Ltd., Beijing 100015, China.

^3^ Experimental and Translational Research Center, Beijing Friendship Hospital, Capital Medical University, Beijing Key Laboratory of Tolerance Induction and Organ Protection in Transplantation & National Clinical Research Center for Digestive Diseases, Beijing 100050, China.

^4^ Division of Liver Transplantation Surgery, Department of Surgery, Beijing Friendship Hospital, Capital Medical University & National Clinical Research Center for Digestive Diseases, Beijing 100050, China.

^5^ Department of Cell Therapy in Regenerative Medicine, University of Tokyo Hospital, Tokyo 113-8655, Japan.

**Methods**

**Histology and immunohistochemistry**

Formalin-fixed liver tissue was embedded in paraffin and cut into 5-μm sections. Liver sections stained with haematoxylin and eosin (HE) were used for necrosis evaluation. For terminal deoxynucleotidyl transferase-mediated dUTP nick-end labelling (TUNEL) analysis, liver sections were stained with a One Step TUNEL Apoptosis Assay Kit (TRITC, Meilunbio, Dalian, China) according to the manufacturer’s instructions. Briefly, after standard dewaxing, the sections were digested by proteinase K (20 μg/ml) in PBS at room temperature for 30 min. After three washes with PBS, each section was incubated with 50 μl TdT Enzyme and TRITC-dUTP Labeling Mix at a ratio of 1:9 for 1 h at room temperature. The nuclei were counterstained with 4’,6-diamidino-2-phenylindole (DAPI; Sigma-Aldrich, St. Louis, MO, USA).

**Liver-infiltrating immune cells**

Nonparenchymal cells in the liver were isolated by collagenase digestion and density centrifugation to analyse liver-infiltrating immune cells. Briefly, liver tissue was perfused with PBS to wash out blood cells in the circulatory system in situ, cut into small pieces for suspension in 10 ml Hanks’ balanced salt solution (HBSS) with 0.1 mg/ml collagenase D (Roche Diagnostics, Mannheim, Germany), 0.01 mg/ml DNase I (Roche Diagnostics), and 0.1 mg/ml BSA (Sigma-Aldrich) and incubated at 37°C for 30 min. Then, the mixture was dissociated with a gentle MACS dissociator (Miltenyi Biotec, Bergisch-Gladbach, Germany). The cell suspension was filtered through a 70-μm nylon cell strainer and centrifuged at 50 × g for 5 min. After discarding the hepatocyte pellet, the supernatant was centrifuged at 500 × g for 5 min to obtain nonparenchymal cells. The nonparenchymal cells were resuspended in 5 ml 30% Percoll-HBSS and centrifuged at 500 × g for 5 min twice. After washing with MACS buffer (PBS containing 2 mmol/l EDTA and 0.5% BSA), the nonparenchymal cells were resuspended in 1 ml erythrocyte lysates (BioLegend) for 5 min on ice. After adding PBS to terminate the effects of erythrocyte lysate and centrifuging at 500 × g for 5 min, 1 μl mouse TruStain fcX (BioLegend) was added to the nonparenchymal cells and incubated on ice for 10 min. Then, the cells were suspended with 100 μl 2% FBS-PBS containing 1 μl of Brilliant Violet-421-labelled rat anti-mouse CD45 antibodies (BioLegend), 1 μl of PerCP5.5-labelled rat anti-mouse Ly-6G antibodies (BioLegend), 1 μl of PE-Cy7-labelled rat anti-mouse CD11b antibodies (BioLegend), 1 μl of APC-labelled rat anti-mouse Ly-6C antibodies (BioLegend), Brilliant Violet 605-labelled rat anti-mouse F4/80 antibodies (BioLegend), and incubated at 4°C for 30 min. After three washes with PBS, the cells were fixed with 1% paraformaldehyde-PBS and analysed on a FACSAria II flow cytometer (BD Biosciences) using CellQuest software (BD Biosciences). The data were analysed by FlowJo software (Treestar, Ashland, OR, USA).

**Cell viability of APAP-treated L-O2 cells and MSCs**

L-O2 cells and MSCs were plated in 96-well plates at 5×10^4^ cells/well and cultured overnight. Medium containing different concentrations of APAP (0 mM, 5 mM, 10 mM, 15 mM, 20 mM, and 25 mM) was added to the cells in eight replicate wells. After 24 h of culture, 3-[4,5-dimethylthiazole-2-yl]-2,5-diphenyltetrazolium bromide (MTT) was added to each well at a final concentration of 0.5 mg/ml and incubated for 4 h. Then, the supernatant was discarded, and DMSO was added to dissolve the formazan crystals. The absorbance at 590 nm was measured using a SpectraMax M5 microplate reader (Thermo).

**Results**


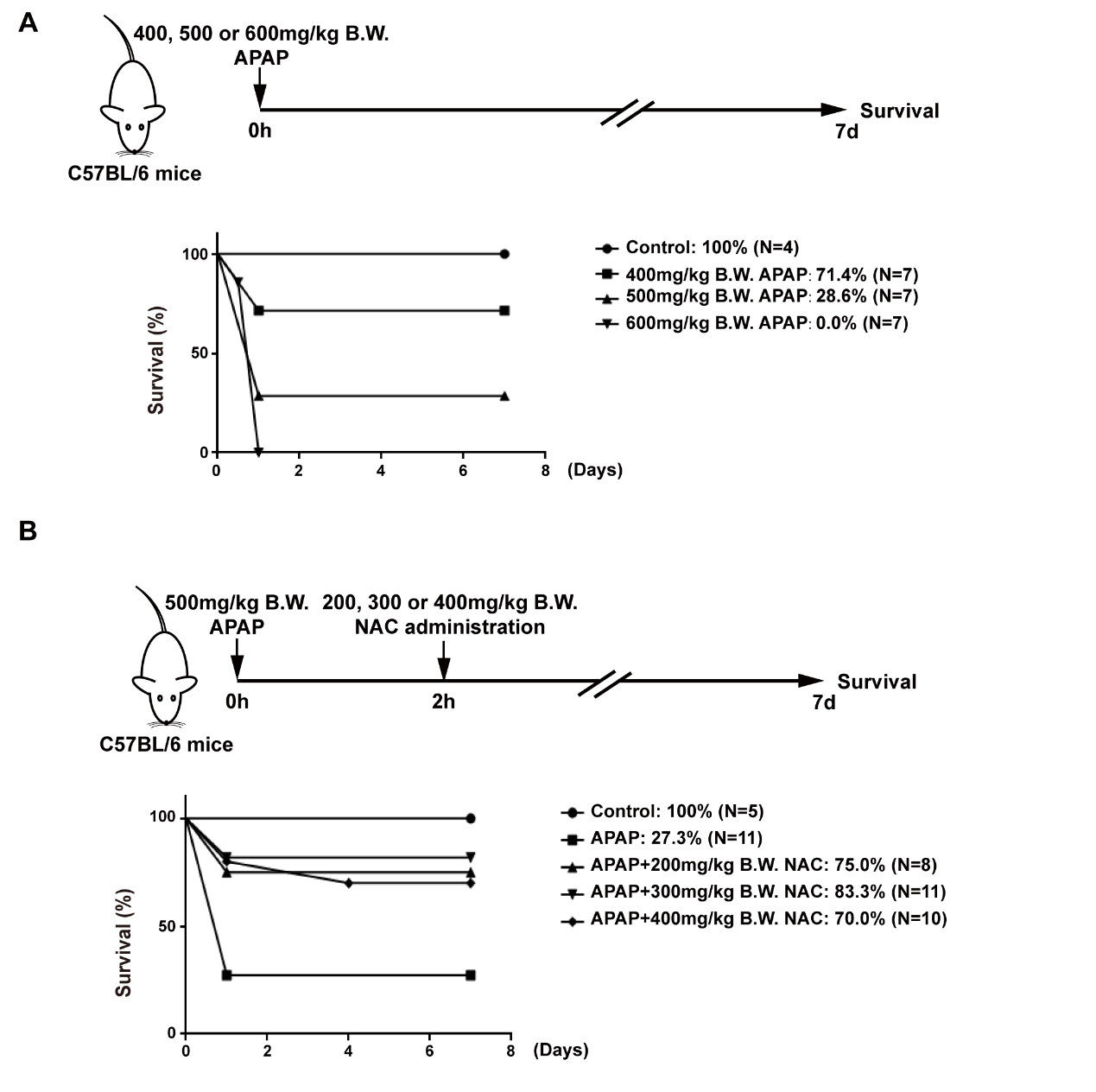


**Fig. S1 Optimize the dose of APAP for liver failure and the dose of NAC for treating APAP-induced liver failure.** **A** Schematic representation of the experiment design and seven-day survival curve to determine the dosage for APAP-induced liver failure in mice. **B** Schematic representation of the experiment design and seven-day survival curve to optimize the NAC dosage to treat APAP-induced liver failure in mice.


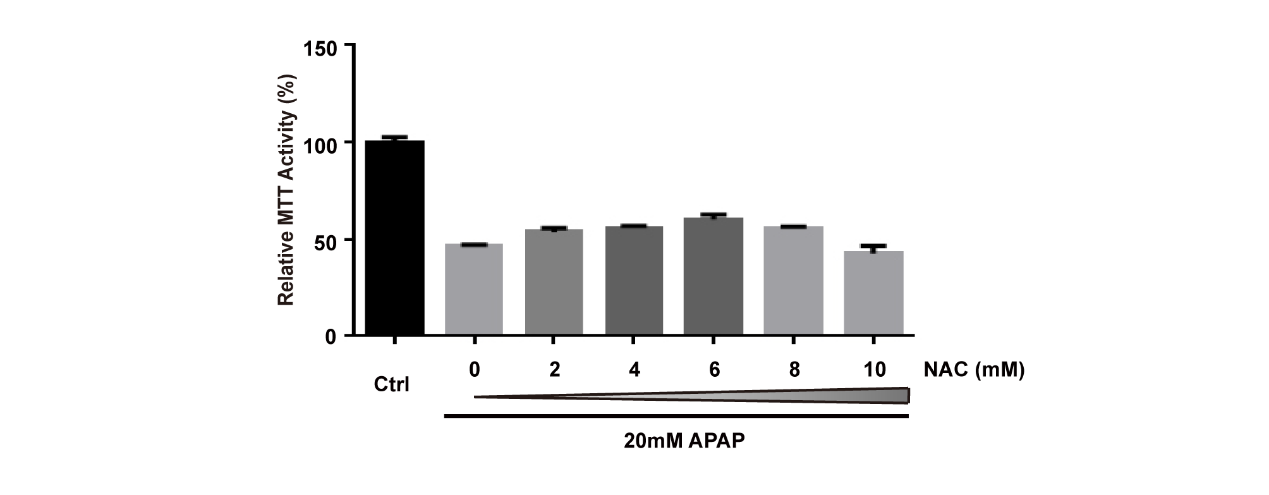


**Fig. S2** **MTT analysis to optimize the NAC dose for blocking APAP-induced L-O2 cell necrosis.**
